# Chemokine control of HIV-1 infection: Beyond a binding competition

**DOI:** 10.1186/1742-4690-7-86

**Published:** 2010-10-13

**Authors:** Yuntao Wu

**Affiliations:** 1 Department of Molecular and Microbiology, George Mason University, Manassas, VA 20110, USA

## Abstract

A recent paper by Cameron *et al. *demonstrated that certain chemokines such as CCL19 activate cofilin and actin dynamics, promoting HIV nuclear localization and integration into resting CD4 T cells. Apparently, these chomokines synergize with the viral envelope protein, triggering cofilin and actin dynamics necessary for the establishment of viral latency. This study opens a new avenue for understanding chemokine interaction with HIV. Traditionally, chemokine control of HIV infection focuses on competitive binding and down-modulation of the corecptors, particularly CCR5. This new study suggests that a diverse group of chemokines may also affect HIV infection through synergistic or antagonistic interaction with the viral coreceptor signaling pathways.

## Introduction

Despite the success of highly active antiretroviral therapy (HAART) in inhibiting HIV replication, viral latency and low-level replication permit viral persistence [1]. HIV can be stably maintained in a variety of cells such as macrophages and resting CD4 T cells. In particular, the long-lived, resting memory CD4 T cells have been shown to be a major viral reservoir. Nevertheless, little is known about the establishment of HIV latency in resting CD4 T cells in the body. Previous studies have suggested that HIV infection of resting CD4 T cells *in vitro *can lead to viral DNA synthesis, although at a slower speed [2,3]. The virus is also capable of mediating nuclear migration with the help of the viral envelope protein that triggers signal transduction to promote cofilin and actin activities [4,5]; viral DNA integration did not occur or was observed at an extremely low level. Because non-integrated viral DNA is not stable, the establishment of a long-term reservoir in resting T cells requires stable integration that normally does not occur in the absence of T cell activation or cytokine stimulation.

The lack of understanding of viral latency in resting T cells has prompted a search for possible cellular conditions that permit viral integration and latency. In 2007, Lewin's group identified a novel mechanism of HIV latent infection of resting CD4 T cells, in which the CCR7 ligands, CCL19 and CCL21, were found to drastically increase the permissiveness of resting CD4 T cells to HIV infection [6]. Specifically, this enhancement was attributed to CCL19/CCL21-mediated increases of viral DNA nuclear migration and integration, but not productive viral replication [6]. Recently, the same group further demonstrated that the molecular mechanism of the CCL19-CCR7 interaction shares similarity with that of the HIV gp120-CXCR4 interaction in triggering cofilin activation and actin dynamics which drastically enhance viral nuclear migration and integration [7]. Apparently, the CXCL19-mediated chemokine signaling synergizes with the gp120-mediated activation of cofilin through the chemokine receptors CCR7 and CXCR4, respectively. Indeed, this appears to be consistent with *in vivo *data showing that in HIV-infected patients, enhanced levels of CCL19 and CCL21 correlate with viral load, disease progression and patients' response to HAART.

These findings open an avenue to examine the role of chemokines in controlling HIV infection, and suggest a potential new way of treating HIV infection. Traditionally, chemokine control of HIV infection focuses on competitive inhibition of viral entry through binding to the chemokine co-receptors, CCR5 in particular. This new result suggests that HIV infection could also be affected with chemokines interacting with multiple receptors such as CCR7, CXCR3, or CCR6 [7] that may synergize or antagonize with HIV-mediated coreceptor signaling pathways. Thus, a much broader range of surface receptors and intracellular signaling molecules could be targeted.

## Main text

**Figure 1:**
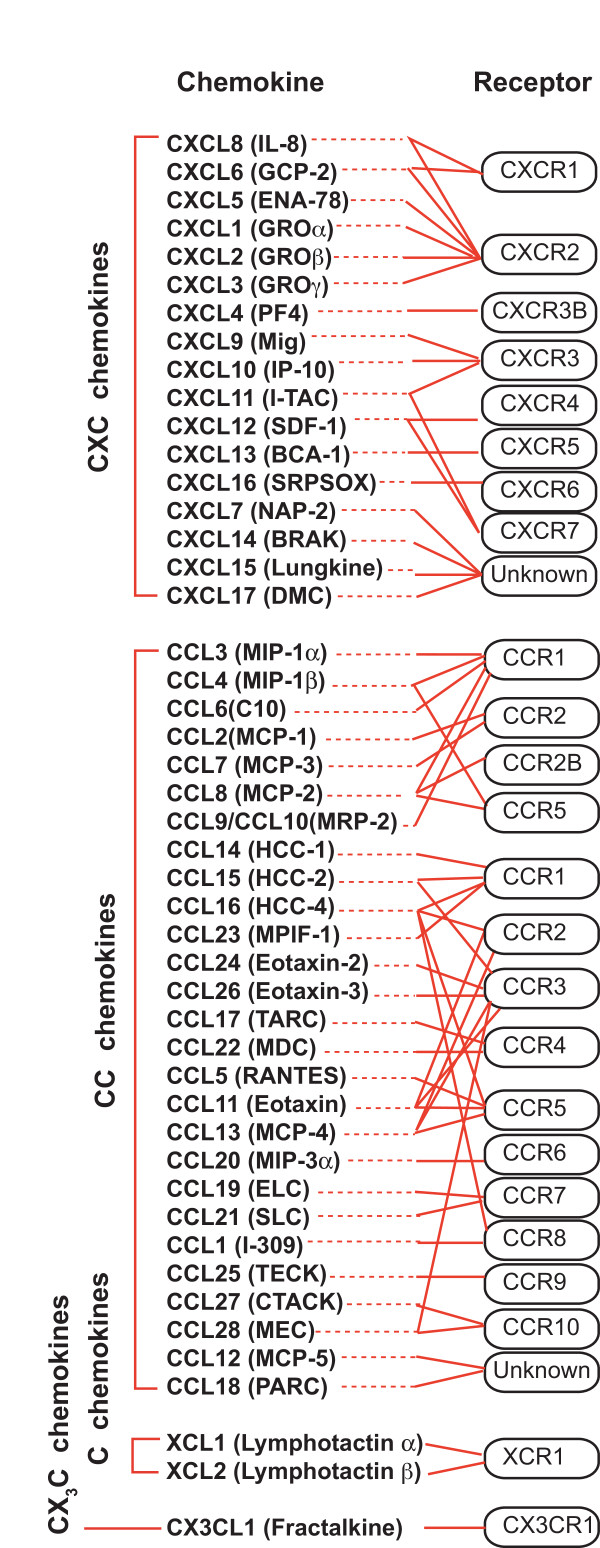
**Human chemokines and their receptors**.

Chemokines are a group of small proteins with chemoattractant properties, promoting leukocyte movement through binding to G-protein-coupled chemokine receptors (GPCR). Currently there are approximately 50 chemokines and 20 receptors identified (Figure 1). Among them are the two main chemokine co-receptors of HIV-1, CXCR4 and CCR5. Binding of chemokines to their cognate GPCRs activates a diverse array of signal pathways. Most of the signaling molecules are components of the signaling transduction pathways mediating chemotactic responses for cytoskeleton rearrangement, cell polarization and migration, as well as transcriptional activation, cell survival and proliferation [8]. Consistent with the signaling diversity of the chemokine-receptor interaction, binding of HIV-1 envelope (gp120) to CCR5 or CXCR4 has also been shown to trigger the activation of multiple intracellular molecules such as cofilin that increases the cortical actin dynamics to facilitate viral nuclear migration [4,8].

In a recent study by Cameron *et al*., the relationship between HIV infection and multiple chemokines was examined. Several key features emerged: (1) Certain chemokines such as CCL19, CXCL9/CXCL10, and CCL20 promote HIV nuclear migration and integration, whereas others such as CCL1 and CCL13 do not. (2) There are only limited changes in gene expression following chemokine exposure, suggesting that the enhancement on HIV infection may not be at the gene expression level. (3) The chemokine enhancement is not associated with T cell activation, as no changes in surface expression of CD69, HLA-DR, and CD25 were observed. (4) Chemokine enhancement only occurs before or at the time of HIV infection, and it is transit (as little as 3 h after treatment) and reversible (lost if removed for more than 3 h), which is consistent with the plasticity of cellular signal transduction, and suggests that the enhancement likely resulted from rapid changes in signaling pathways rather than from breaking cellular restriction factors.

Although cofilin was identified in this study as the key signaling molecule responsible for the CCL19-mediated enhancement, for the chemokine system as a whole, there are likely multiple mechanisms to affect HIV infection, as chemokines are frequently pleiotropic. The Cameron study also suggested possible new ways of controlling HIV infection. Chemokines may be classified into either "synergizer", "antagonist", or "neutral" based on their relationship with HIV infection. Treatment of target cells with chemokine "synergizers" would enhance HIV infection, whereas treatment with an "antagonist" would do the opposite. "Neutral" chemokines may not affect HIV infection in a significant manner.

HIV may also be inhibited through different strategies: (1) through inhibitors that target certain chemokine receptors on the surface. These inhibitors may include either inhibitory antibodies, small-molecule antagonists, nonfunctional chemokines that bind but do not activate viral-dependent pathways, or chemokine antagonists that bind and transduce inhibitory signals for HIV replication; (2) through inhibitors that directly target the intracellular chemokine signaling molecules such as those regulating actin dynamics; (3) through inhibitors that target the down stream effector molecules of chemokine signaling, mainly the cytoskeletal actin that is involved in HIV entry, reverse trancription and nuclear migration [4,9,10].

## Conclusions

The recent transformative study by Cameron *et al. *calls for an expansion of research scope on chemokine control of HIV infection. It is imperative to initiate a systematical investigation into the chemokine signaling network in relation to HIV infection. This would pave the way for future development of new classes of anti-HIV inhibitors that could potentially act at multiple steps along the chemokine signaling pathways.

## Competing interests

The author declares that they have no competing interests.
